# Direct and indirect effects of dominant plants on ecosystem multifunctionality

**DOI:** 10.3389/fpls.2023.1117903

**Published:** 2023-03-02

**Authors:** Jingwei Chen, Ziyang Liu, Hanwen Cui, Hongxian Song, Jiajia Wang, Haining Gao, Shuyan Chen, Kun Liu, Zi Yang, Yajun Wang, Xiangtai Wang, Xiaoli Yang, Lihua Meng, Lizhe An, Sa Xiao, Yoann Le Bagousse-Pinguet

**Affiliations:** ^1^ State Key Laboratory of Grassland and Agro-ecosystems, College of Ecology, Lanzhou University, Lanzhou, Gansu, China; ^2^ Ministry of Education Key Laboratory of Cell Activities and Stress Adaptations, School of Life Sciences, Lanzhou University, Lanzhou, Gansu, China; ^3^ College of Life Science and Engineering, Hexi University, Zhangye, Gansu, China; ^4^ Aix Marseille Univ, Centre national de la recherche scientifique, Avignon Université, Institut de Recherche pour le Développement, Institut Méditerranéen de Biodiversité et d’Écologie marine et continentale, Technopôle Arbois-Méditerranée, Aix-en-Provence, France

**Keywords:** ecosystem multifunctionality, plant biodiversity, soil biodiversity, dominant plants, alpine meadow

## Abstract

Biodiversity is essential for the provision of multiple ecosystem functions simultaneously (ecosystem multifunctionality EMF). Yet, it remains unclear whether and how dominant plant species impact EMF. Here, we aimed at disentangling the direct from indirect above- and belowground pathways by which dominant plant species influence EMF. We evaluated the effects of two dominant plant species (*Dasiphora fruticosa*, and the toxic perennial plant *Ligularia virgaurea*) with expected positive and negative impacts on the abiotic environment (soil water content and pH), surrounding biological communities (plant and nematode richness, biomass, and abundance in the vicinity), and on the EMF of alpine meadows, respectively. We found that the two dominant plants enhanced EMF, with a positive effect of *L. virgaurea* on EMF greater than that of *D*. *fruticosa*. We also observed that dominant plants impacted on EMF through changes in soil water content and pH (indirect abiotic effects), but not through changes in biodiversity of surrounding plants and nematodes (indirect biotic pathway). Our study suggests that dominant plants may play an important role in promoting EMF, thus expanding the pervasive mass-ratio hypothesis originally framed for individual functions, and could mitigate the negative impacts of vegetation changes on EMF in the alpine meadows.

## 1 Introduction

Biodiversity is essential for the provision of multiple ecosystem functions simultaneously (ecosystem multifunctionality; EMF, Hector and Bagchi, 2007), such as litter decomposition, ecosystem production, food web stability, and climate regulation (Van Der Heijden et al., 2008; Bodelier, 2011; Bardgett and Van Der Putten, 2014; Handa et al., 2014). While multiple facets of biodiversity can impact on EMF (e.g. Flynn et al., 2011; Soliveres et al., 2016; Le Bagousse-Pinguet et al., 2019; Le Bagousse-Pinguet et al., 2021; Yuan et al., 2021), focusing solely on diversity *stricto sensu* ignores the overwhelming influence of species dominance on ecosystem functions (Grime, 1998; Garnier et al., 2004). According to the mass-ratio hypothesis (Grime, 1998), the effects of plant species on ecosystem functioning are proportional to plant biomass (Grime, 1998). However, this hypothesis was originally proposed for single functions (Grime, 1998; Smith and Knapp, 2003; Garnier et al., 2004), but yet remains less clear when focusing on EMF (Le Bagousse-Pinguet et al., 2019). Determining the key role of dominant species on EMF not only expands our fundamental understanding of the Biodiversity-EMF relationships, but could also help to prioritize relevant biodiversity attributes in conservation programs (Balvanera et al., 2014; Brum et al., 2017).

Dominant species can impact on ecosystem functioning through multiple pathways. On the one hand, dominant species and their traits can affect directly individual ecosystem functions, such as biomass production or nutrient cycling (e.g. Garnier et al., 2004). For instance, species assemblages dominated by recalcitrant species (i.e. the dominant species that exhibit high leaf lignin concentration) have been shown to decrease EMF, particularly the functions related to decomposition processes (Austin and Ballare, 2010; Le Bagousse-Pinguet et al., 2021). On the other hand, dominant species could also have indirect effects on ecosystem functioning, i.e. through the changes in local above- and belowground diversities. Dominant plant species can have negative effects on plant growth and establishment (e.g. due to asymmetric light competition, Grime, 1973), decreasing local plant biodiversity (Hejda et al., 2021), and ultimately ecosystem functioning (Livingstone et al., 2020). However, these species could also have positive effects (i.e. facilitation, Bertness and Callaway, 1994), and play as ecosystem engineers that enhance abiotic conditions (e.g. soil water content and pH) in their vicinity (Cavieres et al., 2014; Ellison, 2019). In these cases, dominant plants could promote plant (Michalet et al., 2006; Le Bagousse-Pinguet et al., 2014) and soil biodiversity and activity, including soil bacterial (Hortal et al., 2015), and fungal diversity and abundance (Delgado-Baquerizo et al., 2016b; Le Bagousse-Pinguet et al., 2021). Disentangling the direct (positive and negative) from indirect above- and belowground pathways by which dominant plant species influence EMF remains poorly explored, although such an integrative framework could contribute to the global understanding of the impact of biodiversity on ecosystem functioning.

Finally, the mediating role of soil nematodes in the influence of dominant plants on EMF has rarely been explored. Nematodes include a wide variety of trophic groups, such as herbivores, omnivores, predators, and microbial feeders, and are known to play important roles in soil food webs (Ferris, 2010). Furthermore, dominant plants can play a key role in regulating soil nematode abundances (De Deyn et al., 2004) or their richness through changes in understory plant and microbial diversity (Wang et al., 2019b). Altogether, soil nematodes represent key organisms influencing ecosystem functioning, e.g. by grazing on plant roots (Yeates et al., 2009) or by regulating microbial communities, litter decomposition, and nutrient cycling (Yeates et al., 2009; García-Palacios et al., 2016; Yeates and Coleman, 2021). Yet, exploring the impacts of soil nematodes on EMF and their role in the indirect pathways by which dominant plants influence EMF is essential for maintaining high levels of ecosystem function in the context of grassland vegetation change.

Previous studies have shown that soil pH and water content are important drivers of soil biological communities (Fierer and Jackson, 2006), and ecosystem functions or processes (e.g. soil organic carbon accumulation, nitrogen mineralization, and plant productivity) (Chen et al., 2013; Jing et al., 2015; Li et al., 2017). Therefore, here we investigated the role of dominant plant species on the abiotic environment (soil pH and water content), on biological communities (surrounding plants, nematodes, and microbial richness, abundances, and biomasses), and on the EMF of grassland ecosystems using nine soil functions related with biological productivity, nutrient cycling, and build-up of nutrient pools. We focused on alpine grasslands because they cover 86% of the Qinghai-Tibet Plateau (Wang and Cheng, 2001), are biodiversity hot-spots (Bengtsson et al., 2019), and support the provision of essential ecosystem services such as animal husbandry, forage production or carbon sequestration (Clough et al., 2014; Newbold et al., 2016). We used two common plant species with potentially negative and positive effects on biodiversity and EMF: *Ligularia virgaurea* Mattf. ex Rehder. & Kobuski. (*Ligularia* Cass, *Asteraceae*) and *Dasiphora fruticosa* (L.) Rydb. (*Dasiphora*, *Rosaceae*). *L. virgaurea* is a perennial herb widely distributed in the alpine meadows of the Qinghai-Tibet Plateau, and it is a poisonous weed plant (Wang et al., 2008) that can be fatal for animals (Ma et al., 2006; Shi et al., 2011). *D. fruticosa* is a common shrub of the alpine meadows within an elevation range from 2700 to 4500 m.a.s.l (Wang et al., 2017). *D. fructicosa* has been shown to promote the survival of surrounding graminoids (Xu et al., 2010; Michalet et al., 2014) or through indirect effects that promote nematode abundance by increasing grass biomass (Wang et al., 2018). However, these positive effects can be hidden by complex indirect interactions among plant functional groups (Xu et al., 2010; Michalet et al., 2014). We hypothesized that: (1) *D. fruticosa* would increase and *L. virgaurea* would reduce the EMF; (2) dominant plants would influence EMF through modifying the abiotic drivers, such as soil water content and pH; (3) dominant species would also indirectly affect EMF by influencing biodiversity, especially soil nematodes.

## 2 Materials and methods

### 2.1 Study site

The experiment was conducted in the alpine grasslands of the eastern Qinghai-Tibet Plateau, i.e. at the Gansu Gannan Grassland Ecosystem National Observation and Research Station in Maqu (33°40′N, 101°51′E) at 3550 m.a.s.l, Gansu, China. The mean annual temperature is 1.2°C, with the lowest temperatures occurring in January (-10°C) and the highest in July (11.7°C). The mean annual precipitation reaches 564 mm, mostly concentrated from May to September. Yaks have been grazing the alpine grassland in the study area since 1999, with a density of 1.6 head ha^-1^ (Hu et al., 2015). Vegetation did not experience significant degradation over the least 20 years. The vegetation cover of the study site is dominated by the shrub *Dasiphora fruticosa* (Rosaceae) and *Ligularia virgaurea* (Asteraceae), but also includes other perennial plant species such as *Carex atrofusca* (Cyperaceae), *Agrostis hugoniana* (Poaceae), *Euphorbia altotibetica* (Euphorbiaceae) and *Halenia elliptic*a (Gentianaceae) (See **Supplementary Table 1** for more information about each plant species).

### 2.2 Experimental design

Our observational design was set up in early June 2016 (See **Supplementary Figure 1**). Fifteen blocks were set up within a homogenous and flat alpine grassland landscape, which is one of the representative landscape types of the Tibetan Plateau and the main typical habitat of the distribution of *D. fruticosa* and *L. virgaurea* (Chu et al., 2014; Qian et al., 2022). Two hundred individuals of *D. fruticosa* could be observed (25% of the total cover), and thousands of *L. virgaurea* (15% of the cover) in this landscape. Each block included 3 plots of 30 cm × 30 cm: one grassland plot including the dominant shrub *D. fruticosa*, one including the dominant poisonous weed *L. virgaurea* and one grassland control without *D. fruticosa* or *L. virgaurea*. The canopy size of each shrub was around 50 cm × 70 cm in our study, so to ensure that the sample plot was completely under the canopy, we set up a 30 cm × 30 cm plot. The dominant plant represented the center of the plot. Altogether, our study included a total of 45 plots.

### 2.3 Sampling and measurement

In each plot, we collected a composite soil sample, resulting from three sub-samples (4 cm diameter) randomly taken by a soil auger at 15 cm depth. We mixed each composite soil sample and removed the gravel. We divided the composite soil samples into two replicates, and stored them at 4°C: one replicate was used to extract nematodes, and the other to measure soil physicochemical properties.

We measured 9 soil variables that were uncorrelated with each other (See **Supplementary Figure 2**), and together constitute good proxies for biological productivity, nutrient cycling, and nutrient pools establishment (Bowker et al., 2013; Soliveres et al., 2016; Delgado-Baquerizo et al., 2016a; Wang et al., 2019a): soil nitrate (NO_3_^-^), soil ammonium (NH_4_^+^), soil organic carbon (SOC), soil total phosphorus (TP), soil total nitrogen (TN), urease (URE), phosphatase (PHO), invertase (INV), protease (PRO).

We measured soil water content (SWC) by taking 5 g fresh soil and placing them in an oven at 90°C for 48 h until constant weight. After removing plant residues and gravel, the remaining soil was air-dried (avoiding direct sunlight), and then sieved (aperture of 0.25 mm). Soil pH was quantified in a 1:2.5 soil: deionized water slurry using a pH meter (PHSJ-3F, Shanghai INESA Scientific Instrument Co., Ltd, China). TP and TN were digested by concentrated H_2_SO_4_, followed by Mo-Sb antispetrophotography and semi micro-Kjeldahl (Bao, 2000) with an auto chemistry analyzer (SmartChem 200, AMS Alliance, Italy). SOC was determined following the wet oxidation method. Soil NH_4_^+^ and NO_3_^-^ were extracted using 2 M KCl (1:10 soil: solution ratio) and analyzed with an auto chemistry analyzer.

The activity of URE was measured by the reaction of urease enzyme ammonia with phenol-sodium hypochlorite in an alkaline medium (Huang et al., 2015). The activity of INV was measured by using the dinitrosalicylic acid method (Asare-Brown and Bullock, 1988). PHO activity was determined by using disodium diphenyl phosphate colorimetry (Tabatabai and Bremner, 1969). Ninhydrin colorimetry was used to determine PRO activity (Watanabe and Hayano, 1995).

### 2.4 Biodiversity indices

We focused on plant species richness, biomass, and abundance as potential aboveground indirect biotic drivers, as they are known to have an impact on EMF (e.g. Maestre et al., 2012; Soliveres et al., 2016). In each plot, all herbaceous plants were thus identified at the species level to calculate the plant richness. We also counted the number of each individual plant species to evaluate the abundance of aboveground plant communities and then harvested per species to assess the aboveground biomasses. The plant material was oven-dried for 48 h at 80 ° before weighing. Note that the Shannon index of plant diversity was also calculated. However, this index was further removed from subsequent analyses due to high correlation with plant richness (r = 0.8, **Supplementary Figure 3**).

We also considered the richness, biomass, and abundance of nematodes as potential indirect soil biotic drivers of dominant plant species on EMF. We used the modified Baermann wet funnel technique to extract nematodes from 50 g. of fresh soil (Liu et al., 2008). We identified all nematodes in each sample to genus/species level and converted nematode abundances to the number of individuals per kg. of dry soil (ind. kg^-1^ dry soil). We also calculated the Shannon index of nematode diversity. However, this index was further removed from subsequent analyses due to high correlation with nematode richness (r = 0.9, **Supplementary Figure 3**). We finally measured the maximum width and length of all nematodes observed. We used Andrassy’s formula (Andrássy, 1967) to estimate nematode biomass:



*eq. 1*, $${\text{Weight}}_{\text{nematode}}\left(\text{μg}\right)=\frac{{\text{W}}^{2}\text{(μm)}}{1{.6\times 10}^{6}}\times \text{L (μm)}$$

where W is the maximal width of nematodes and the L is their length.

Finally, we considered microbial C and N biomasses as proxies of the C and N sources in the systems (Sanaullah et al., 2011). They were measured based on the Chloroform fumigation extraction method (Brookes et al., 1985). Then a microbial C:N biomass ratio was calculated and used as a surrogate of ecosystem productivity and soil fertility (Sanaullah et al., 2011; Cheng et al., 2020).

### 2.5 Multifunctionality indices

We evaluated EMF using both the averaging (Mouillot et al., 2011) and the multiple threshold approaches (Byrnes et al., 2014), which allowed us to assess whether multiple functions are simultaneously performing at high levels, and to consider potential trade-offs between the functions assessed (Le Bagousse-Pinguet et al., 2019; Le Bagousse-Pinguet et al., 2021). The averaged EMF index (EMF_A_) was calculated using each of the 9 soil variables (SOC, TN, TP, NH_4_^+^, NO_3_^-^, URE, PRO, PHO, INV). We scaled the 9 variables to range from 0 to 1 with the formula:



*eq.2*, $$f\left(x\right)=\frac{x_{i}-x_{min\;}}{x_{max}-x_{min}}$$

where *x* is the value of the function with its maximum (*x*_max_) and minimum (*x*_min_) values measured, then we averaged the standardized variables to obtain the EMF_A_ for each plot. We also computed EMF-threshold values of 25% (MF_T25_), 50% (MF_T50_), 75% (MF_T75_), according to Byrnes et al. (2014).

### 2.6 Statistical analyses

Generalized linear mixed modeling (GLMM) was used to evaluate the effects of *D. fruticosa*, *L. virgaurea*, and the control on EMF, and on plant and nematode diversity and biomass and soil properties, with the treatments as fixed effects, and block as a random effect. The uniformity and dispersion of these models were checked and adjusted accordingly. The beta and poisson generalized linear models were used for proportional and counting data, respectively (Douma and Weedon, 2019). We also used “compois” or “genpois” distribution due to the presence of underdispersion (i.e. variance < mean) in the data (Zuur et al., 2007). Tukey’s HSD test was used for post-hoc analyses to determine significant differences between treatments.

Linear mixed effect models (LMM) were used to evaluate the impacts of abiotic and biotic drivers on EMF. We used the EMF indices as response variables, and soil (pH, SWC, and their quadratic term) and biotic attributes (dominant plants; plant richness, abundance, and biomass; nematode richness, abundance, and biomass; microbial biomass C:N) as predictors, and included the block as a random effect. Note that since some diversity index do not necessarily change linearly along environmentally strong gradients (e.g. soil pH and water content), we considered the quadratic term of soil pH and water content. Before regression analysis, the predictors highly correlated (r > 0.7) and the variance inflation factor (VIF) value of more than 10, such as plant and nematode Shannon diversity, were removed from all analyses (See **Supplementary Figure 3** and **Supplementary Table 2**).

All response variables and predictors were Z-scored (standardized deviated) prior to analyses to account for parameter estimates within a comparable scale. To assess the relative effect of each predictor on EMF, we used a method similar to the variance decomposition. In short, the method can be simply calculated the ratio between the standardized regression coefficients of predictors and the sum of all standardized regression coefficients in the models (Gross et al., 2017; Yuan et al., 2021). We also repeated these analyses without random effect to ensure the robustness of our results (See **Supplementary Table 3**).

For each EMF index, a model selection procedure was used to select the most parsimonious set of predictors (Le Bagousse-Pinguet et al., 2017). We first generated all possible combinations of predictors, and then selected the set of best-supported models within a ΔAICc of 2 (See **Supplementary Table 4**). Before analysis, we scaled all predictors using the Z-scored (standardized deviated) method (Le Bagousse-Pinguet et al., 2017).

Finally, piecewise structural equation modeling (pSEM) was used to test for the direct and indirect effects of *L. virgaurea* and *D. fruticosa* on EMF through changes in abiotic and biotic attributes. We set up an a-priori models, while only considering dominant plants, and the significant biotic and abiotic indirect drivers found with the linear modeling procedure (**Supplementary Figure 5**). We thus considered the dominant plant type *D. fruticosa* or *L. virgaurea)* as predictors, SWC and pH as indirect abiotic drivers, and plant and nematode richness as indirect biotic drivers of EMF. The model fits of pSEM were assessed using Shipley’s test of d-separation through Fisher’s *C* statistic (Lefcheck, 2016).

Our data analyses were conducted in R software, ver. 4.0.3 (R core Team, 2020). The calculation of EMF indices was conducted using the *getStdAndMeanFunctions* function in the ‘multifunc’ package (Byrnes et al., 2014). Shannon diversity was calculated using the *diversity* function in the ‘vegan’ package (Oksanen et al., 2019). The GLMMs were performed using the *glmmTMB* function with a genpois link (i.e. count data for the underdispersion), or with a beta link (i.e. proportional data) in the ‘glmmTMB’ package (Brooks et al., 2017) and the *lmer* (i.e. normal data) and *glmer* function with a poisson link (i.e. count data for no dispersion) in the ‘lme4’ package (Bates et al., 2015). The model diagnosis of linear mixed models was conducted using the *testUniformity* function in the ‘DHARMa’ package (Hartig, 2020), and the *testDispersion* function in the ‘DHARMa’ package (Hartig, 2020) for the dispersion test. The *dredge* function in the ‘MuMIn’ package (Bartoń, 2020) for the model selection procedure. The marginal means (EMMS) of GLMMs and LMMs were estimated using the *emmeans* function in package ‘emmeans’ (Lenth et al., 2020). The pSEMs were conducted using the *psem* function in the R package ‘piecewiseSEM’ (Lefcheck, 2016). Package ‘ggplot2’ (Wickham, 2016) was used to plot figures.

## 3 Results

**Figure 1:**
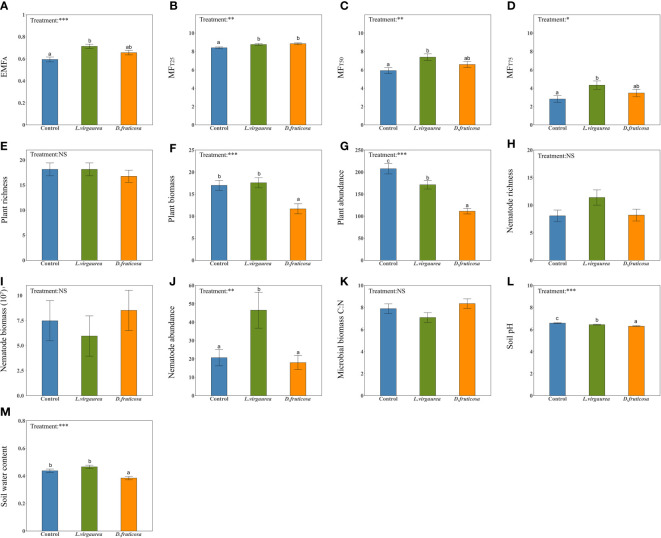
The effects of dominant plants on EMF_A_ **(A)**, MF_T25_ **(B)**, MF_T50_ **(C)**, MF_T75_ **(D)**, plant richness **(E)**, plant biomass **(F)**, plant abundance **(G)**, nematode richness **(H)**, nematode biomass **(I)**, nematode abundance **(J)**, Microbial biomass C:N ratio **(K)**, soil pH **(L)**, soil water content **(M)**. Different lowercase letters within panels indicate significant (*p*-value < 0.1) differences between treatment means, after using Tukey’s method to correct for multiple comparisons. Error bars represent means ± SE (NS, *p*> 0.05; *p <0.05; **p <0.01; ***p <0.001).

EMF indices strongly varied in response to dominant plants (**Figure 1**). The allopathic *L. virgaurea* significantly increased all EMF indices compared to the control plots (**Figures 1A–D**). This was particularly true for individual functions such as SOC, TN, NH4^+^, and for most of the enzymes considered (See **Supplementary Figures 4A, C, E–I**). The facilitative species *D. fruticosa* mostly led to intermediate values of EMF indices (**Figures 1A, C, D**), although a significant increase in EMF was observed when the ecosystem was performing low (MF_T25_: **Figure 1B**). This pattern mostly occurred because the effect of *D. fruticosa* on individual ecosystem functions were not consistent, and could be either positive (SOC, TN, NH4^+^, INV) (See **Supplementary Figures 4A, C, E, F**) or neutral (TP, NO_3_^-^, URE, PHO) (See **Supplementary Figures 4B, D, H, I**).

We found that dominant plants had contrasted effects on the indirect biotic and abiotic drivers considered (**Figures 1E–M**). *D. fruticosa* significantly decreased plant biomass and abundance (**Figures 1F, G**). *L.virgaurea* had a negative effect on plant abundance (**Figure 1G**), a positive effect on nematode abundance (**Figure 1J**), and a neutral effect on plant biomass (**Figure 1F**) compared to the control plots. In contrast, dominant plants had no impact on the richness of understory plant and nematode (**Figures 1E, H**), nematode biomass (**Figure 1I**), and microbial biomass C:N ratio (**Figure 1K**). Finally, dominant plants had significant effects on the abiotic attributes considered, particularly by significantly decreasing the soil pH (**Figure 1L**). *D. fruticosa* furthermore had a significant negative effect on SWC, while SWC under *L. virgaurea* remained as high as in the control plots (**Figure 1M**).

**Figure 2:**
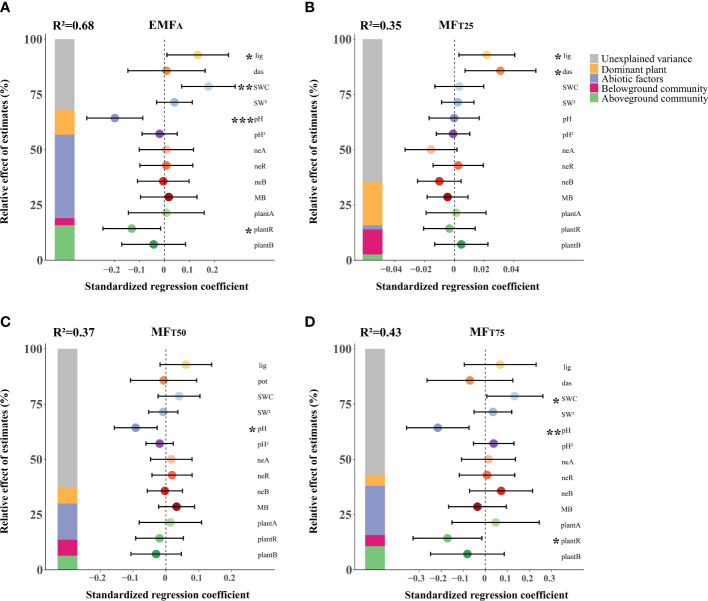
The effects of biotic and abiotic factors on EMF_A_ **(A)**, MF_T25_ **(B)**, MF_T50_ **(C)**, MF_T75_ **(D)**. Standardized regression coefficients of model predictors, associated 95% confidence intervals and relative importance of each factor, expressed as the percentage of explained variance. The R^2^ of the averaged model and the *p*-value of each predictor are given as: *p < 0.05; **p < 0.01; ***p < 0.001. lig, *L. virgaurea*; das, *D. fruticosa*; SWC, soil water content; pH, soil pH; neA, nematode abundance; neR, nematode richness; neB, nematode biomass; MB, microbial biomass; C, N ratio; plantA, plant abundance; plantR, plant richness; plantB, plant biomass.

The multiple linear regression models explained a fair amount of variation in EMF, i.e. ~68%, 35%, 37%, and 43% of variations in EMF_A_, MF_T25_, MF_T50,_ and MF_T75_ respectively (**Figure 2**). The dominant plants *D. fruticosa* and *L. virgaurea* together explained on average ~11% of the variation in EMF (5%~20%). In comparison, the abiotic attributes considered explained ~20% of the variations in EMF (2~38%), and the cumulative above- and belowground attributes accounted for ~9% (3%~16%) and 7% (3%~11%) of the variations in EMF respectively.

*L. virgaurea*, SWC, soil pH, and plant richness were the main predictors of EMF (**Figure 2A**). However, these effects were highly dependent on the level of performance of the system. *D. fruticosa* and *L. virgaurea* positively affected EMF at the low level of ecosystem performance (MF_T25_). Plant richness had a negative effect on EMF, specifically at the higher level of EMF (MF_T75_). Increasing SWC enhanced EMF, specifically at the higher level of performance (MF_T75_), and soil pH also reduced EMF in the case of higher levels of ecosystem performance (MF_T50_ and MF_T75_).

**Figure 3:**
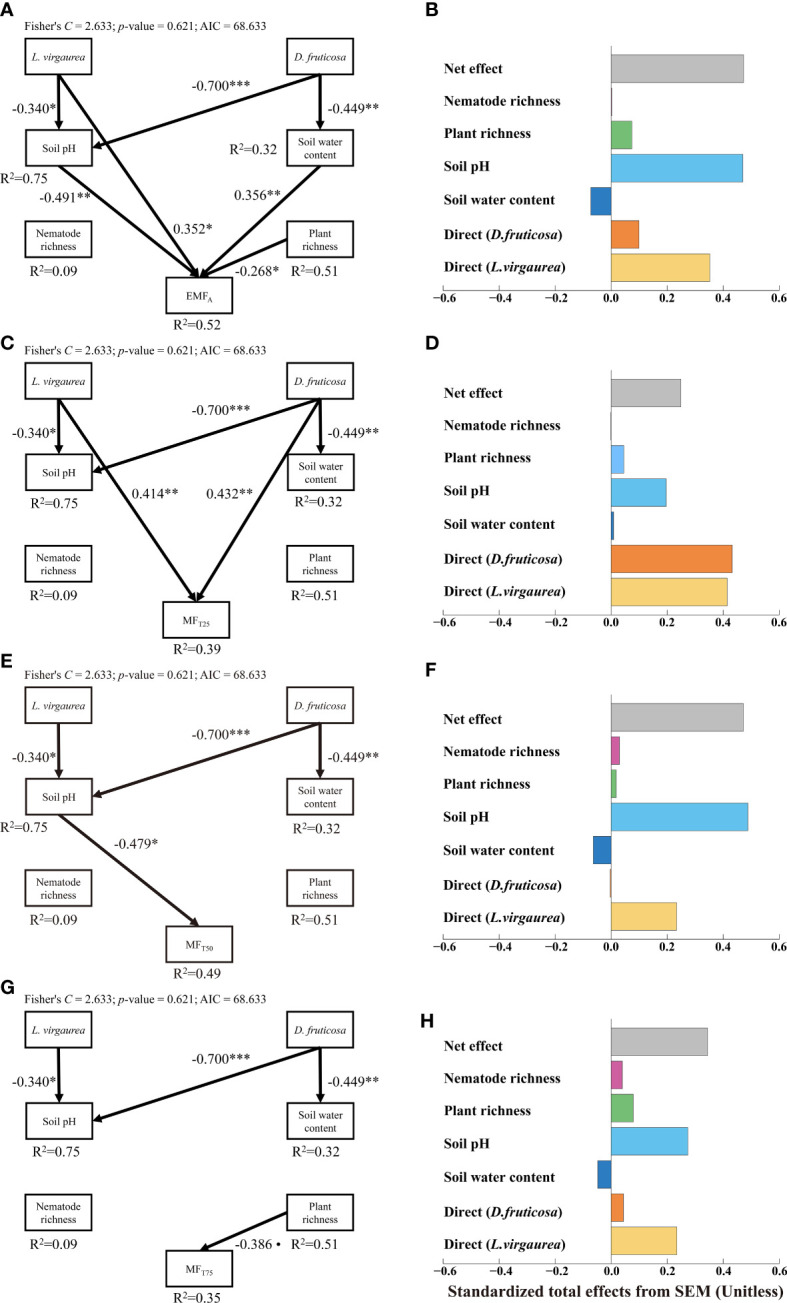
Structural equation model assessing the direct and indirect effects of dominant plants on EMF_A_ **(A)**, MF_T25_ **(C)**, MF_T50_ **(E)** and MF_T75_ **(G)**. Numbers adjacent to arrows are indicative of the effect size of the relationship. Only significant and marginally significant pathways were shown. Significance levels are as follows: •, *p* < 0.10; *p < 0.05; **p < 0.01; ***p < 0.001. The right panel of the figure showed that total, direct and indirect standardized effects of the different drivers of EMFA **(B)**, MF_T25_ **(D)**, MF_T50_ **(F)** and MF_T75_ **(H)**.

The pSEMs explained 52%, 39%, 49%, and 35% of the variations in EMF_A_, MF_T25_, MT_T50_, and MF_T75_ respectively (**Figure 3**). From the pSEMs results, we can find that the impacts of *D. fruticosa* and *L. virgaurea* on EMF were both a promotion effect, irrespective of the EMF threshold considered, and the promotion effect of *L. virgaurea* was greater than that of *D. fruticosa* (except MF_T25_). The pSEMs showed that *L. virgaurea* can directly enhance EMF_A_ and MF_T25_, and *D. fruticosa* significantly improved MF_T25_ directly. Meanwhile, both *D. fruticosa* and *L. virgaurea* indirectly affected the EMF through the abiotic pathway. The pSEMs also showed indirect effects of *L. virgaurea* on EMF_A_ and MF_T50_ *via* soil pH (**Figures 3A, E**), while *D. fruticosa* influenced EMF_A_ indirectly through soil pH and SWC (**Figure 3A**). We also considered other biological pathways, such as nematode and plant abundance (See **Supplementary Figure 6**), nematode, plant, and microbial biomass (See **Supplementary Figure 7**). The results also showed that the dominant plants affected EMF mainly through the direct path and abiotic indirect path, and the promotion effect of *L. virgaurea* on EMF was higher than that of *D. fruticosa*.

## 4 Discussion

Here we aimed at disentangling the direct from indirect abiotic and biotic (above- and belowground diversities) pathways by which dominant plant species influence EMF. Our results indicated that the two dominant plants overall promoted the EMF of alpine grasslands. Our result brings new evidence on the importance of dominant plants for EMF and thus expands to multiple ecosystem functions simultaneously the mass-ratio hypothesis (Grime, 1998), originally framed for individual functions (Smith and Knapp, 2003; Garnier et al., 2004).

The positive effect of dominant plants on EMF occurred irrespective of their expected (positive and negative) effects. Two main reasons may explain this pattern. First, dominant plants can alter the spatial allocation of resources (e.g. fertilizer island effects) (Maestre and Puche, 2009; Eldridge et al., 2011; Ochoa-Hueso et al., 2018). For instance, Soliveres and Eldridge (2014) found that increasing shrub encroachment did not impede ecosystem functions, but instead had positive effects on plant and soil properties. Second, grazing may have played an important role in this process (Daryanto et al., 2013; Soliveres and Eldridge, 2014). Upon herbivore trampling effects on soil compaction and water redistribution that could reduce nematode diversity and ecosystem functioning (Castellano and Valone, 2007; Allington and Valone, 2010; Andriuzzi and Wall, 2017), the unexpected facilitative effect of *L. virgaurea* on EMF may arise from mechanisms of associational avoidance (see Milchunas and Noy-Meir, 2002 for a review). On the other hand, and according to the attractant decoy hypothesis (Milchunas and Noy-Meir, 2002), herbivores preferentially consume palatable plants, such as grasses, thereby altering the aboveground plant community composition of grasslands (control) and affecting nutrient input and decomposition (Abule et al., 2005; see Hempson et al., 2015 for a review). We acknowledge that our approach does not allow us to fully conclude on the mechanisms explaining the observed patterns, but this overall positive effect clearly stimulates the need for further research on the impact of dominants on EMF.

Contrary to the first hypothesis, we did not observe any positive effects of *D. fruticosa* on EMF indices (except MF_T25_), although it is often regarded as a nurse species (Michalet et al., 2016; Wang et al., 2017). Our result may relate to the environmental conditions under which our experimental design was performed. Species interactions can shift from competitive to facilitative interactions from low to moderate stress environmental conditions (Bertness and Callaway, 1994). Facilitation then diminishes from moderate to highly stressed conditions (Michalet et al., 2006; Michalet et al., 2014). In this context, and depending on the environmental conditions, the dominant plant *D. fruticosa* could have various effects on local species diversity (Le Bagousse-Pinguet et al., 2012; Liancourt et al., 2017).

The positive effects of *L. virgaurea* on EMF were greater than that of *D. fruticosa*, to significantly increase each component of EMF (e.g. soil organic carbon, total nitrogen, ammonium, and for most of the enzymes considered). *L. virgaurea* has been found to increase total soil organic carbon concentrations, soil organic C:N ratio, and enzymatic activity (Shi et al., 2011), or root secretions to promote multiple bacterial groups (Wang et al., 2022). Also, leaching from allelopathic plants such as *L. virgaurea* can also improve soil conditions in their vicinity (Hierro and Callaway, 2003). However, our results do not call for their expansion as a potential standardization of management. Previous studies have also found that the expansion of toxic weeds could reduce grassland areas (Van Auken, 2009), thus limiting the access to palatable food sources for herbivores (Ma et al., 2006). Therefore, and while beneficial to soil functions, this species may have negative effects on key functions on which human society depends. These antagonistic effects warn for the need of comprehensive efforts when formulating management policies to deal with the expansion of toxic weeds rather than a ‘one size fits all’ management approach.

Our results also showed that dominant plants mostly impacted on EMF through changes in soil pH and water content. Soil pH is known to be an important driver impacting on microbial communities (Lauber et al., 2009) and EMF (Delgado-Baquerizo et al., 2016b; Luo et al., 2018). Furthermore, soil pH was highly related to soil organic carbon, and soil acidification could contribute to soil organic carbon accumulation, thereby improving the ability of grassland ecosystems to maintain multiple functions (Jing et al., 2015). Soil water content had an indirect positive effect on ecosystem functions, which may relate to the increase of water holding capacity, further promoting nutrient cycling and grassland productivity (Guo et al., 2012).

Contrary to expectation, we found negligible indirect biotic effects, indicating that dominant plants weakly affected the EMF of alpine grasslands through changes in the richness of plants and key soil organisms such as nematodes. Furthermore, the effects of plant richness were only observed at high levels of ecosystem performance. Our results align with the view of considering other facets of biodiversity such as functional and phylogenetic diversity facets, which have been found to contribute more to EMF than taxonomic richness only (Flynn et al., 2011; Gross et al., 2017; Le Bagousse-Pinguet et al., 2019; Wen et al., 2019; Le Bagousse-Pinguet et al., 2021). Also, the absence of effect of belowground biodiversity may arise from the consideration of nematodes only as an indicator of belowground biodiversity, while multiple trophic levels are needed to promote EMF (Jing et al., 2015; Schuldt et al., 2018). The reason may be that the soil functions (e.g. soil enzymes) we selected may be more related to abiotic factors (e.g. soil pH and water content) than to nematode diversity. Studies on the relationship between soil species richness and ecosystem function also show that for nutrient cycling, it depends to some extent on species traits rather than species richness (Heemsbergen et al., 2004; Nielsen et al., 2011). In addition, there may be trade-offs in the relationship between nematodes of different feeding types and EMF. For example, Du et al. (2022) found that bacterial feeders were positively correlated with EMF, while fungal feeders and omnivorous feeders were negatively correlated with EMF.

## 5 Conclusions

Our findings provided evidence that contrasting dominant plants such as *D. fruticosa* and *L. virgaurea* can increase ecosystem multifunctionality in the alpine meadows, although our “one-shot” study should be complemented by longer-term and dynamical approaches. Finally, our results also showed that these effects not only arise from direct, but also indirect abiotic pathways through changes in soil conditions. Altogether, our study suggested that dominant plants may play a key role in promoting multiple ecosystem functions simultaneously, and could mitigate the negative impacts of vegetation changes on EMF in the alpine meadows.

## Data availability statement

The original contributions presented in the study are included in the article/**Supplementary Material**. Further inquiries can be directed to the corresponding author.

## Author contributions

JC, SC, HG, KL, LA, and SX contributed to conception and design of the study. ZL, HC, HS, JW, ZY, YW, XW, XY, and LM conducted the field experiment and organized the database. JC performed all the statistical analyses. JC and YB-P wrote the manuscript. All authors contributed to the article and approved the submitted version.

## Supplementary material

Supplementary Figure 1 The schematic diagram of experimental design.

Supplementary Figure 2 Pearson’s correlation coefficients between each individual function and their relationships with EMF indices. Red to black color indicates negative to positive correlations. The number represent correlation coefficient. TP: soil total phosphatase; TN: soil total nitrogen; SOC: soil organic carbon; NO_3_^-^: soil nitrate; NH_4_^+^: soil ammonium; INV: invertase; PRO: protease; URE: urease; PHO: phosphatase; EMF_A_: averaged multifunctionality index; MF_T25_: 25% threshold-based multifunctionality index; MF_T50_: 50% threshold-based multifunctionality index; MF_T75_: 75% threshold-based multifunctionality index.

Supplementary Figure 3 Pearson’s correlation coefficients between pairs of abiotic and biotic factors. Red to black color indicates negative to positive correlations. The number represent correlation coefficient. lig: *L. virgaurea*; das: *D. fruticosa*; SWC: soil water content; pH: soil pH; plantA: plant abundance; plantR: plant richness; plantB: plant biomass; plantS: plant Shannon diversity. neA: nematode abundance; neR: nematode richness; neB: nematode biomass; neS: nematode Shannon diversity; MB:microbial biomass C:N ratio.

Supplementary Figure 4 The effects of dominant plants on each of ecosystem functions. Different lowercase letters within panels indicate significant (NS: *p*> 0.05; *: *p <*0.05; **: *p <*0.01; ***: *p <*0.001) differences between treatment means, after using Tukey’s method to correct for multiple comparisons. Error bars represent means ± SE. For abbreviations, see **Supplementary Figure 2**.

Supplementary Figure 5 An a-priori conceptual model of piecewise structural equation modeling.

Supplementary Figure 6 Structural equation model assessing the direct and indirect effects of dominant plants on averaged ecosystem multifunctionality **(A)**, 25% threshold-based **(C)**, 50% threshold-based **(E)** and 75% threshold-based **(G)** ecosystem multifunctionality *via* abiotic factors (soil water content and pH) and biotic factors (nematode and plant abundance). Numbers adjacent to arrows are indicative of the effect size of the relationship. Only significant and marginally significant pathways were shown. Significance levels are as follows: •, *p* < 0.10; *, *p* < 0.05; **, *p* < 0.01; ***, *p* < 0.001. The right panel of the figure showed that total, direct and indirect standardized effects of the different drivers of EMF.

Supplementary Figure 7 Structural equation model assessing the direct and indirect effects of dominant plants on averaged ecosystem multifunctionality **(A)**, 25% threshold-based **(C)**, 50% threshold-based **(E)** and 75% threshold-based **(G)** ecosystem multifunctionality *via* abiotic factors (soil water content and pH) and biotic factors (nematode, plant and microbial biomass). Numbers adjacent to arrows are indicative of the effect size of the relationship. Only significant and marginally significant pathways were shown. Significance levels are as follows: •, *p* < 0.10; *, *p* < 0.05; **, *p* < 0.01; ***, *p* < 0.001. The right panel of the figure showed that total, direct and indirect standardized effects of the different drivers of EMF.
