# Supplementary figures and images for: Direct and indirect effects of dominant plants on ecosystem multifunctionality
Source: Front Plant Sci. 2023 Mar 2;14:1117903. doi: 10.3389/fpls.2023.1117903 (PMC10017997; doi:10.3389/fpls.2023.1117903)

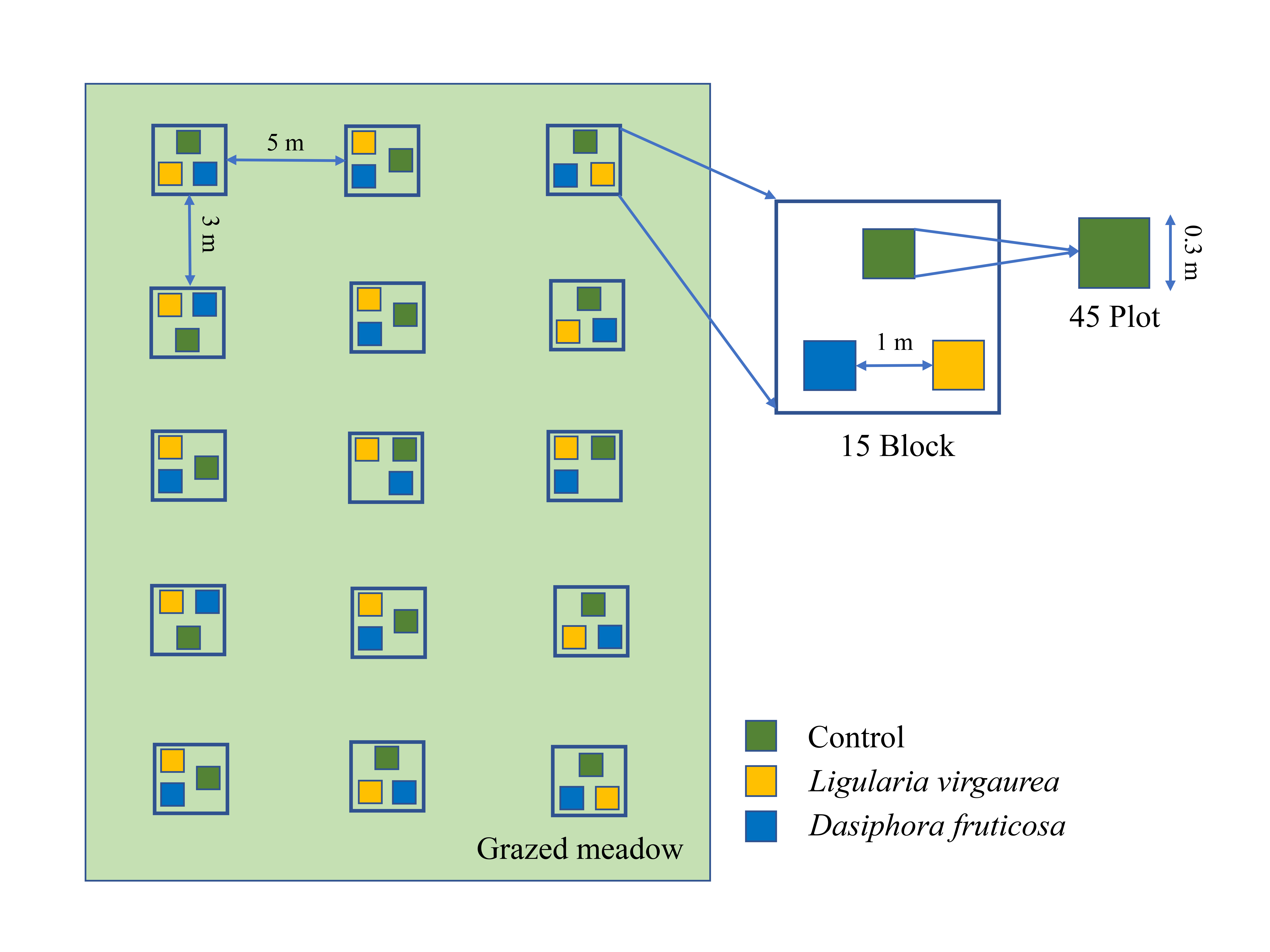

Supplement: Supplementary Figure 1 — The schematic diagram of experimental design. [file Image_1.jpeg]

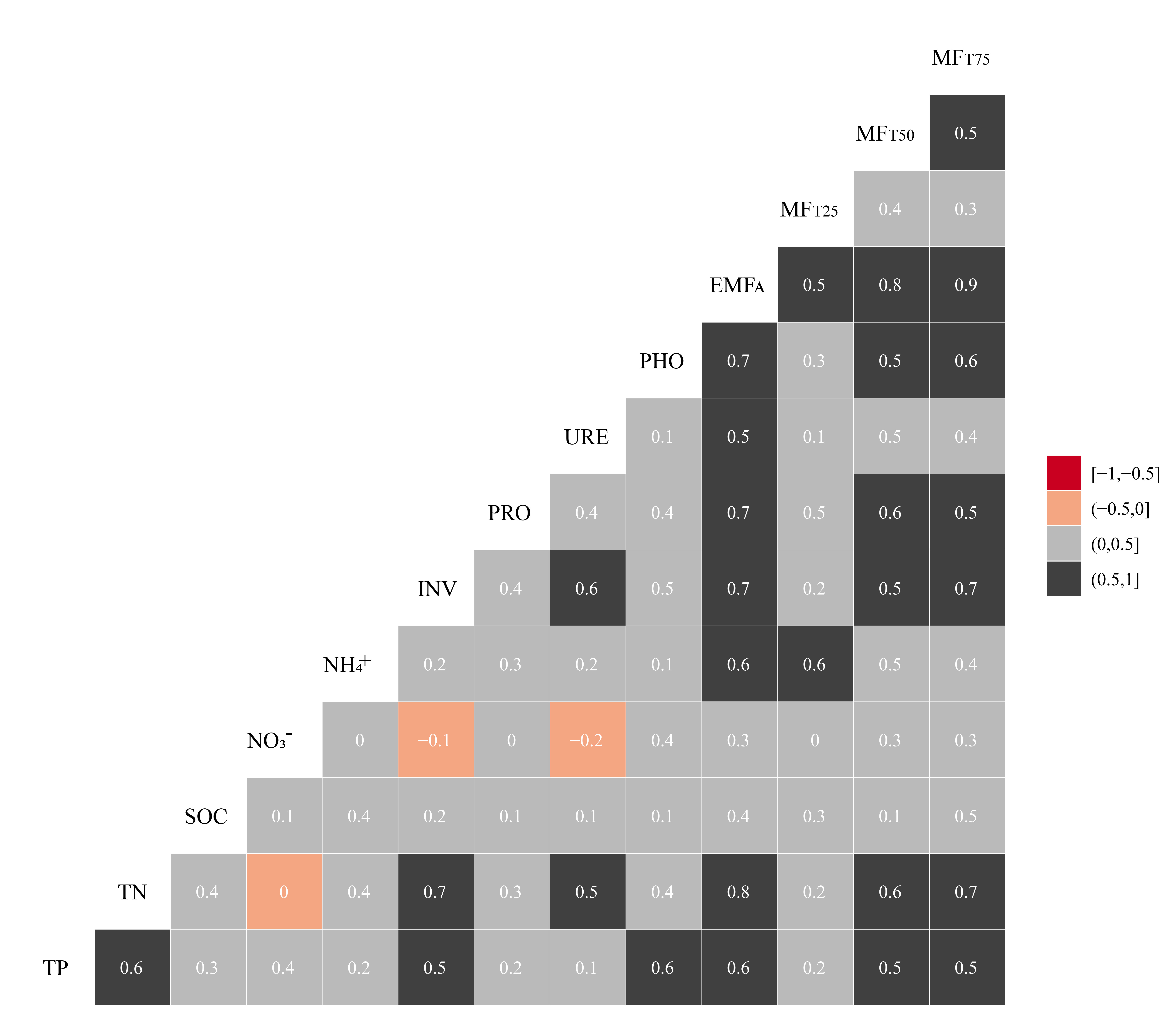

Supplement: Supplementary Figure 2 — Pearson’s correlation coefficients between each individual function and their relationships with EMF indices. Red to black color indicates negative to positive correlations. The number represent correlation coefficient. TP: soil total phosphatase; TN: soil total nitrogen; SOC: soil organic carbon; NO3 -: soil nitrate; NH4 +: soil ammonium; INV: invertase; PRO: protease; URE: urease; PHO: phosphatase; EMFA: averaged multifunctionality index; MFT25: 25% threshold-based multifunctionality index; MFT50: 50% threshold-based multifunctionality index; MFT75: 75% threshold-based multifunctionality index. [file Image_2.jpeg]

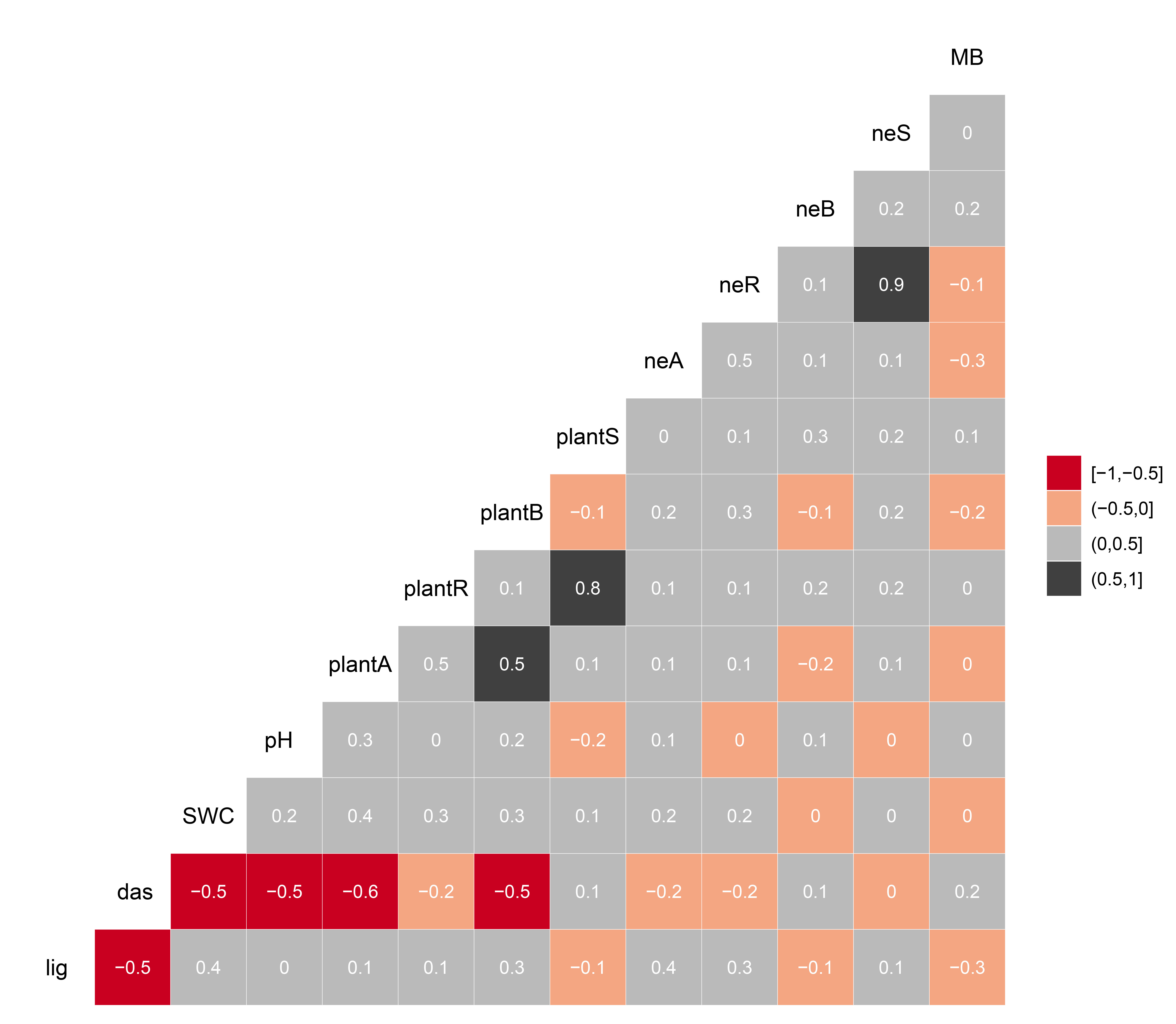

Supplement: Supplementary Figure 3 — Pearson’s correlation coefficients between pairs of abiotic and biotic factors. Red to black color indicates negative to positive correlations. The number represent correlation coefficient. lig: L. virgaurea; das: D. fruticosa; SWC: soil water content; pH: soil pH; plantA: plant abundance; plantR: plant richness; plantB: plant biomass; plantS: plant Shannon diversity. neA: nematode abundance; neR: nematode richness; neB: nematode biomass; neS: nematode Shannon diversity; MB:microbial biomass C:N ratio. [file Image_3.jpeg]
