# Supplementary material for: Direct and indirect effects of dominant plants on ecosystem multifunctionality
Source: Front Plant Sci. 2023 Mar 2;14:1117903. doi: 10.3389/fpls.2023.1117903 (PMC10017997; doi:10.3389/fpls.2023.1117903)
Supplement: Supplementary file 8 [file Table_1.docx]

Supplementary Table 1. A list of plant species identified in the study. The number represent mean ± SE (n = 15) of each plant species abundance.

| Aboveground Plant Species | Group | Control | *L. virgaurea* | *D. fruticosa* |
| --- | --- | --- | --- | --- |
| *Agrostis hugoniana* | Grass | 2.00±0.81 | 5.07±2.37 | 0.93±0.35 |
| *Carex atrofusca* | Grass | 39.30±10.50 | 30.8±5.03 | 11.40±1.90 |
| *Deschampsia caespitosa* | Grass | 0.87±0.56 | 5.67±1.57 | 0.07±0.07 |
| *Elymus nutans* | Grass | 14.33±3.07 | 28.53±4.30 | 7.93±1.93 |
| *Festuca ovina* | Grass | 2.80±0.69 | 1.27±0.46 | 2.47±1.03 |
| *Kobresia capillifolia* | Grass | 2.80±1.28 | 0.87±0.45 | 4.20±1.02 |
| *Kobresia macrantha* | Grass | 1.73±0.93 | 7.47±1.70 | 2.13±0.77 |
| *Koeleria litvinowii* | Grass | 0.00±0.00 | 0.07±0.07 | 0.73±0.73 |
| *Poa pachyantha* | Grass | 2.93±0.89 | 2.67±1.34 | 8.33±2.49 |
| *Poa poophagorum* | Grass | 0.73±0.49 | 2.87±1.15 | 7.13±89 |
| *Scirpus pumilus* | Grass | 27.67±4.79 | 23.33±3.33 | 10.40±2.37 |
| *Astragalus polycladus* | Forb | 0.13±0.13 | 0.00±0.00 | 0.00±0.00 |
| *Oxytropis kansuensis* | Forb | 0.27±0.15 | 0.27±0.15 | 0.13±0.09 |
| *Anaphalis lactea* | Forb | 1.60±0.63 | 0.53±0.47 | 8.93±3.80 |
| *Anemone obtusiloba* | Forb | 11.93±8.14 | 0.07±0.07 | 0.13±0.09 |
| *Anemone rivularis* | Forb | 2.40±1.17 | 0.33±0.21 | 0.27±0.15 |
| *Anemone trullifolia* | Forb | 3.80±1.67 | 0.13±0.13 | 0.00±0.00 |
| *Arenaria serpyllifolia* | Forb | 0.00±0.00 | 0.27±0.15 | 0.33±0.21 |
| *Caltha palustris* | Forb | 1.53±0.70 | 0.53±0.47 | 0.60±0.60 |
| *Cardamine tangutorum* | Forb | 0.07±0.07 | 0.13±0.09 | 1.13±0.61 |
| *Cerastium fontanum* | Forb | 1.00±0.48 | 0.80±0.53 | 0.40±0.29 |
| *Comastoma pulmonarium* | Forb | 0.00±0.00 | 0.00±0.00 | 0.07±0.07 |
| *Draba eriopoda* | Forb | 0.07±0.07 | 0.00±0.00 | 0.40±0.16 |
| *Epilobium palustre* | Forb | 0.07±0.07 | 0.00±0.00 | 0.00±0.00 |
| *Equisetum arvense* | Forb | 0.20±0.11 | 0.27±0.18 | 0.53±0.29 |
| *Euphorbia altotibetica* | Forb | 7.27±2.76 | 8.40±1.65 | 5.53±1.05 |
| *Euphrasia regelii* | Forb | 0.20±0.20 | 0.20±0.20 | 1.13±0.77 |
| *Galium asperuloides* | Forb | 1.2±1.00 | 0.67±0.27 | 2.80±0.96 |
| *Gentiana macrophylla* | Forb | 0.33±0.33 | 0.27±0.27 | 1.40±1.40 |
| *Gentiana sino-ornata* | Forb | 1.33±0.59 | 0.20±0.20 | 0.33±0.23 |
| *Gentiana aristata* | Forb | 0.07±0.07 | 0.53±0.27 | 0.07±0.07 |
| *Gentianopsis paludosa* | Forb | 0.00±0.00 | 0.07±0.07 | 0.07±0.07 |
| *Geranium pylzowianum* | Forb | 1.27±0.67 | 0.93±0.40 | 0.00±0.00 |
| *Halenia elliptica* | Forb | 3.33±2.18 | 1.07±0.40 | 1.40±0.83 |
| *Juncus effusus* | Forb | 0.07±0.07 | 0.07±0.07 | 0.80±0.24 |
| *Lamiophlomis rotata* | Forb | 1.27±0.69 | 2.00±1.80 | 0.00±0.00 |
| *Lancea tibetica* | Forb | 8.00±1.25 | 5.20±1.28 | 2.80±0.94 |
| *Leontopodium souliei* | Forb | 8.93±2.05 | 6.60±3.49 | 4.33±1.92 |
| *Lomatogonium carinthiacum* | Forb | 1.27±0.61 | 0.40±0.19 | 0.33±0.23 |
| *Parnassia trinervis* | Forb | 0.87±0.46 | 0.27±0.12 | 0.80±0.44 |
| *Pedicularis kansuensis* | Forb | 0.33±0.23 | 0.00±0.00 | 0.00±0.00 |
| *Plantago depressa* | Forb | 5.27±1.64 | 0.73±0.30 | 0.73±0.73 |
| *Pleurospermum camtschaticum* | Forb | 0.53±038 | 5.20±1.67 | 1.40±0.60 |
| *Polygonum viviparum* | Forb | 3.57±1.67 | 3.47±1.38 | 3.07±1.40 |
| *Potentilla anserina* | Forb | 28.87±4.62 | 17.53±1.80 | 6.27±1.56 |
| *Potentilla saundersiana* | Forb | 6.53±2.78 | 0.80±0.49 | 0.93±0.87 |
| *Ranunculus tanguticus* | Forb | 2.20±0.86 | 0.67±0.43 | 0.33±0.19 |
| *Saussurea hieracioides* | Forb | 0.13±0.13 | 0.53±0.41 | 0.20±0.11 |
| *Saussurea stella* | Forb | 2.07±1.03 | 1.40±1.19 | 0.93±0.80 |
| *Saxifraga pseudohirculus* | Forb | 0.07±0.07 | 0.20±0.20 | 3.33±1.68 |
| *Scrofella chinensis* | Forb | 0.13±0.09 | 0.73±0.67 | 0.13±0.13 |
| *Silene aprica* | Forb | 0.00±0.00 | 0.00±0.00 | 0.20±0.14 |
| *Taraxacum mongolicum* | Forb | 0.47±0.26 | 0.00±0.00 | 0.47±0.40 |
| *Thalictrum alpinum* | Forb | 1.20±0.87 | 2.07±1.36 | 2.53±1.55 |
| *Trigonotis peduncularis* | Forb | 2.13±0.93 | 1.33±0.50 | 0.40±0.29 |
| *Viola pseudo-bambusetorum* | Forb | 4.87±3.90 | 1.47±0.39 | 2.00±0.83 |
