# Supplementary material for: Direct and indirect effects of dominant plants on ecosystem multifunctionality
Source: Front Plant Sci. 2023 Mar 2;14:1117903. doi: 10.3389/fpls.2023.1117903 (PMC10017997; doi:10.3389/fpls.2023.1117903)
Supplement: Supplementary file 9 [file Table_2.docx]

Supplementary Table 2. Results of the variance inflation factor (VIF) to evaluate the risk of multicollinearity. VIFs are calculated for each predictor for multifunctionality. VIF values < 10 indicate that there is no collinearity. For abbreviations, see Fig.S2 and S3, pH^2^: the quadratic term of soil pH; SWC^2^: the quadratic term of soil water content.

| Mod | EMF~lig+das+pH+pH^2^+SWC+SWC^2^+neA+neR+neB+plantA+plantR+plantB+MB | | | | | | | | | | | | |
| --- | --- | --- | --- | --- | --- | --- | --- | --- | --- | --- | --- | --- | --- |
| Variable | lig | das | pH | pH^2^ | SWC | SWC^2^ | neA | neR | neB | plantA | plnatR | plantB | MB |
| EMF_A_ | 2.511 | 4.089 | 1.991 | 1.500 | 1.773 | 1.548 | 1.647 | 1.789 | 1.382 | 3.682 | 2.003 | 2.495 | 1.493 |
| MF_T25_ | 2.496 | 3.913 | 1.857 | 1.498 | 1.752 | 1.557 | 1.598 | 1.781 | 1.400 | 3.787 | 2.114 | 2.563 | 1.463 |
| MF_T50_ | 2.601 | 3.960 | 1.815 | 1.462 | 1.848 | 1.670 | 1.606 | 1.770 | 1.383 | 3.747 | 2.176 | 2.568 | 1.519 |
| MF_T75_ | 3.083 | 4.359 | 1.857 | 1.485 | 2.058 | 1.980 | 1.626 | 1.818 | 1.379 | 4.409 | 2.443 | 2.908 | 1.539 |
