# Supplementary material for: Direct and indirect effects of dominant plants on ecosystem multifunctionality
Source: Front Plant Sci. 2023 Mar 2;14:1117903. doi: 10.3389/fpls.2023.1117903 (PMC10017997; doi:10.3389/fpls.2023.1117903)
Supplement: Supplementary file 10 [file Table_3.docx]

Supplementary Table 3. The results of multiple regression models for EMF, with and without considering “block” as a random effect. For abbreviations, see Fig.S2.

|  | EMF_A_ | MF_T25_ | MF_T50_ | MF_T75_ |
| --- | --- | --- | --- | --- |
|  | AIC | AIC | AIC | AIC |
| With random effect | -94.324 | 71.486 | 169.120 | 179.750 |
| Without random effect | -96.316 | 69.486 | 167.120 | 178.420 |
| anova test (*p*-value) | 0.930 | 0.999 | 0.999 | 0.413 |
