# Supplementary material for: Direct and indirect effects of dominant plants on ecosystem multifunctionality
Source: Front Plant Sci. 2023 Mar 2;14:1117903. doi: 10.3389/fpls.2023.1117903 (PMC10017997; doi:10.3389/fpls.2023.1117903)
Supplement: Supplementary file 11 [file Table_4.docx]

Supplementary Table 4. The results of multiple regression models for EMF. We tested the effects of biotic and abiotic factors on EMF. We provided Model R^2^, AICc, ΔAICc (< 2) and weight for the selected models. For abbreviations, see Fig.S2 and S3, pH^2^: the quadratic term of soil pH; SWC^2^: the quadratic term of soil water content.

|  | lig | das | pH | pH^2^ | SWC | SWC^2^ | neA | neR | neB | MB | plantA | plantR | plantB | R^2^ | AICc | ΔAIC | Weight |
| --- | --- | --- | --- | --- | --- | --- | --- | --- | --- | --- | --- | --- | --- | --- | --- | --- | --- |
| EMF_A_ |  |  |  |  |  |  |  |  |  |  |  |  |  | 0.56 | -105.49 | 0.00 | 0.41 |
|  |  |  |  |  |  |  |  |  |  |  |  |  |  | 0.58 | -105.27 | 0.22 | 0.37 |
|  |  |  |  |  |  |  |  |  |  |  |  |  |  | 0.57 | -104.22 | 1.27 | 0.22 |
| MF_T25_ |  |  |  |  |  |  |  |  |  |  |  |  |  | 0.25 | 58.32 | 0.00 | 0.29 |
|  |  |  |  |  |  |  |  |  |  |  |  |  |  | 0.33 | 58.39 | 0.07 | 0.28 |
|  |  |  |  |  |  |  |  |  |  |  |  |  |  | 0.27 | 58.71 | 0.39 | 0.24 |
|  |  |  |  |  |  |  |  |  |  |  |  |  |  | 0.04 | 59.10 | 0.78 | 0.20 |
| MF_T50_ |  |  |  |  |  |  |  |  |  |  |  |  |  | 0.32 | 153.89 | 0.00 | 0.42 |
|  |  |  |  |  |  |  |  |  |  |  |  |  |  | 0.35 | 154.19 | 0.30 | 0.36 |
|  |  |  |  |  |  |  |  |  |  |  |  |  |  | 0.33 | 155.27 | 1.39 | 0.21 |
| MF_T75_ |  |  |  |  |  |  |  |  |  |  |  |  |  | 0.37 | 168.35 | 0.00 | 0.33 |
|  |  |  |  |  |  |  |  |  |  |  |  |  |  | 0.37 | 169.28 | 0.93 | 0.21 |
|  |  |  |  |  |  |  |  |  |  |  |  |  |  | 0.38 | 169.29 | 0.95 | 0.21 |
|  |  |  |  |  |  |  |  |  |  |  |  |  |  | 0.38 | 170.20 | 1.85 | 0.13 |
|  |  |  |  |  |  |  |  |  |  |  |  |  |  | 0.37 | 170.33 | 1.98 | 0.12 |
